# *Streptococcus mutans* activates the AIM2, NLRP3 and NLRC4 inflammasomes in human THP-1 macrophages

**DOI:** 10.1038/s41368-018-0024-z

**Published:** 2018-08-06

**Authors:** Yuri Song, Hee Sam Na, Eunjoo Park, Mi Hee Park, Hyun Ah Lee, Jin Chung

**Affiliations:** 1 Department of Oral Microbiology, School of Dentistry, Pusan National University, Yangsan, 50162 Korea; 2 Institute of Translational Dental Sciences, Pusan National University, Yangsan, 50162 Korea

## Abstract

*Streptococcus mutans (S. mutans)*, a major aetiologic agent of dental caries, is involved in systemic diseases, such as bacterial endocarditis, if it enters the bloodstream through temporary bacteraemia. Interleukin (IL)-1β, a proinflammatory cytokine, is related to the host defences against pathogens, and its synthesis, maturation, and secretion are tightly regulated by the activation of the inflammasome, an inflammatory signalling complex. This study examined the signalling mechanism of IL-1β secretion and the inflammasome pathway induced by *S. mutans* to explain the molecular mechanism through which systemic infection by oral streptococci can occur. After infection of THP-1 cells with *S. mutans*, the expression of inflammasome components was detected using various methods. *S. mutans* induced IL-1β secretion via caspase-1 activation, and *S. mutans*-induced IL-1β secretion required absent in melanoma (AIM2), NLR family pyrin domain-containing 3 (NLRP3) and NLR family CARD domain-containing 4 (NLRC4) inflammasome activation. In particular, the *S. mutans*-induced NLRP3 inflammasome was mediated by adenosine triphosphate (ATP) release, potassium depletion and lysosomal damage. Our study provides novel insight into the innate immune response against *S. mutans* infection.

## Bacterial infection: The immune response to Streptococcus mutans

*Streptococcus mutans* triggers an early-stage immune response via a chain of events that leads to the activation of immune component, interleukin-1β. A group of academics from Korea’s Pusan National University, led by Jin Chung, tested the response of human immune cell cultures to infection with *S. mutans*—a common oral bacteria that can cause systemic disease if it enters the bloodstream. The group found that S. mutans infection leads to the activation of protein signaling complexes known as inflammasomes, which in turn activate an enzyme known as caspase-1. Caspase-1 then activates interleukin-1β, a pro-inflammatory molecule that forms part of a host’s defenses against pathogens. The team also identified that ATP release, ion depletion, and integrity of intracellular enzyme containers, also impacted inflammasome activation. This study offers insights into the immune response against *S. mutans* and may inform future investigations into systematic oral bacteria infections.

## Introduction

Oral streptococci, referred to as viridans streptococci, account for 20% of all oral bacteria. They include several species, such as *Streptococcus mutans(S. mutans), S. mitis, S. sanguis*, and *S. intermedius*.^1^ Oral streptococci, including *S. mutans*, can enter the bloodstream through temporary bacteraemia in humans after dental extractions, brushing teeth, and even chewing. Temporary bacteraemia can cause bacterial colonisation on heart valve tissues, particularly in patients with pre-existing cardiac valve damage.^2^ After colonisation, the bacterial cells proliferate in the heart valve, which ultimately leads to systemic diseases such as infective endocarditis. Clinically, oral streptococci contribute to infective endocarditis in more than 20% of cases.^3^ Oral streptococcal species, including *S. mutans*, were detected in the heart valves and atheromatous plaques of atherosclerosis patients.^4^ *S. mutans*, a bacterium best known for causing dental caries, expresses a range of virulence factors promoting its invasion into the host cell.^5^ Invasive *S. mutans* induces various inflammatory cytokines in human aortic endothelial cells.^6^ However, the pathogenic mechanism of endocarditis caused by *S. mutans* is not completely understood.

Early inflammation against a bacterial infection is mediated by the innate immune response. Monocytes and macrophages produce inflammatory cytokines to fight bacteria.^7^ Interleukin (IL)-1β is a representative proinflammatory cytokine released by immune cells and is important in the host defence by mediating many responses, including phagocyte activation, antibody production, and T-cell polarisation.^8^ The inflammasome is an intracellular protein complex that controls the activation of caspase-1 and consists of a sensor protein, an adaptor protein, and procaspase-1. The sensor protein is typically a nucleotide-binding oligomerization domain (NOD)-like receptor (NLR) or an absent in melanoma 2 (AIM2) family member.^9^ The NLR and AIM2 family members bind to the adaptor protein called apoptosis-associated speck-like protein containing a caspase recruitment domain (ASC). In turn, the bound proteins recruit procaspase-1 for activation. The NLRs include two prototypical proteins: NLRP3 (also known as NALP3 or cryopyrin^10^) and NLRC4 (also known as IPAF or CARD12^11,12^). When the inflammasome complex is formed in response to various stimuli, activated caspase-1 cleaves pro-IL-1β to its mature and secreted form, IL-1β.^13^ Activation of caspase-1 not only leads to inflammation but also causes an inflammatory form of cell death called pyroptosis.^14^

This study examined the molecular mechanism of IL-1β secretion and the inflammasome pathway in THP-1 cells in response to *S. mutans* challenges to obtain more comprehensive insight into the innate immune response against *S. mutans* infections.

## Results

### *S. mutans* activates caspase-1 and induces IL-1β secretion

**Fig. 1:**
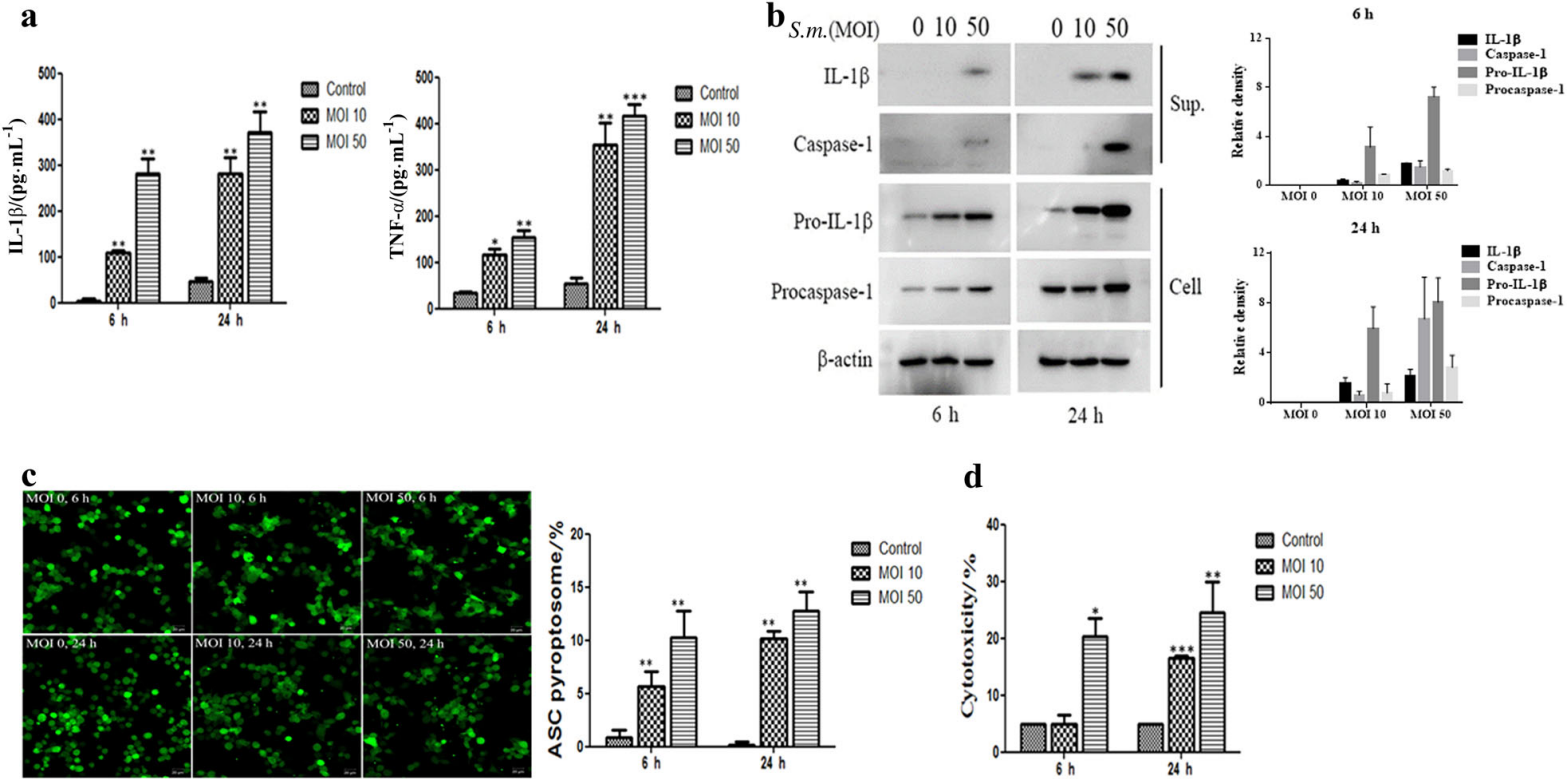
*S. mutans* induces IL-1β secretion and caspase-1 activation, leading to cell death. **a** THP-1 cells were infected with *S. mutans* (MOI 10 or 50) for 6 h or 24 h. The cell culture supernatants were assayed for human IL-1β and TNF-α by ELISA. **b** THP-1 cells were infected with *S. mutans* (MOI 10 or 50) for 6 or 24 h. IL-1β and caspase-1 secreted into the culture supernatants (sup.) and pro-IL-1β, procaspase-1, and β-actin in the cell lysates (cell) were detected by immunoblotting. The relative western blot band densities were normalised to those of β-actin. **c** THP-1/ASC-GFP cells infected with *S. mutans* at each MOI for the indicated times were observed and photographed by fluorescence confocal microscopy (original magnification ×100). The graph in the lower panel shows the percentage of cells containing ASC pyroptosomes. The percentage of cells containing ASC pyroptosomes was calculated as described in the Materials and Methods. **d** The amount of the cytoplasmic enzyme LDH released into the culture supernatant was measured using an LDH cytotoxicity assay kit. The results represent those from one of three individual experiments. The data are reported as the means ±  standard deviations (*n* = 3). *P < 0.05, **P < 0.01, ***P < 0.001. *S.m.* means *S. mutans*

To determine whether *S. mutans* induces inflammation, THP-1 cells were infected with *S. mutans*, and inflammatory cytokine secretion was measured by enzyme-linked immunosorbent assay(ELISA). The levels of IL-1β and TNF-α production increased in a multiplicity of infection (MOI)- and time-dependent manner (Fig. 1a). Based on analysis of the culture supernatants, *S. mutans* activated caspase-1 (Fig. 1b). *S. mutans* also induced the expression of pro-IL-1β and procaspase-1 in the cell lysates. This evidence suggests that *S. mutans* activates caspase-1 and induces pro-IL-1β synthesis and IL-1β secretion in THP-1 cells.

Caspase-1 activation requires the ASC-related pyroptosome, which subsequently mediates inflammatory cell death^14^. Therefore, this study examined whether *S. mutans* induces ASC-dependent pyroptosome formation using THP-1 cells stably expressing an ASC- green fluorescent protein(GFP) fusion protein. Briefly, ASC forms a single 1- to 2-mm supramolecular assembly in macrophage cells against proinflammatory stimuli.^15^ ASC-GFP was distributed in the cytoplasm and nucleus under the usual conditions. After infection of the cells with *S. mutans*, large, bright spots of ASC-GFP fluorescence were recorded in the cytoplasm, indicating the formation of the ASC pyroptosome. Quantitative analysis showed that the percentage of cells that formed pyroptosomes increased in an MOI- and time-dependent manner after *S. mutans* stimulation (Fig. 1c). In addition, *S. mutans-*induced cell death, as measured by the release of lactate dehydrogenase(LHD), increased significantly depending on the MOI and infection time (Fig. 1d). Overall, these results indicate that *S. mutans* induces caspase-1 activation, ASC pyroptosome formation, and IL-1β secretion in THP-1 cells.

### *S. mutans* induces caspase-1-dependent IL-1β secretion and caspase-independent cell death

Many studies have reported that IL-1β release requires the synthesis of pro-IL-1β and its cleavage into mature IL-1β by activated caspase-1. To determine whether *S. mutans*-induced IL-1β secretion is mediated by caspase-1 activation, THP-1 cells were pretreated with benzyloxycarbonyl-V-A-D-O-methyl fluoromethyl ketone (Z-VAD-FMK), a pan-caspase inhibitor, or Z-Trp-Glu(OMe)-His-Asp(OMe)-fluoromethyl ketone (Z-WEHD-FMK), a caspase-1-specific inhibitor, prior to *S. mutans* infection. Inhibiting the caspase pathway with Z-VAD-FMK or Z-WEHD-FMK resulted in the distinct blockade of IL-1β secretion from the *S. mutans*-challenged THP-1 cells. These results suggest that *S. mutans*-induced IL-1β secretion is dependent on caspase-1 activation.

**Fig. 2:**
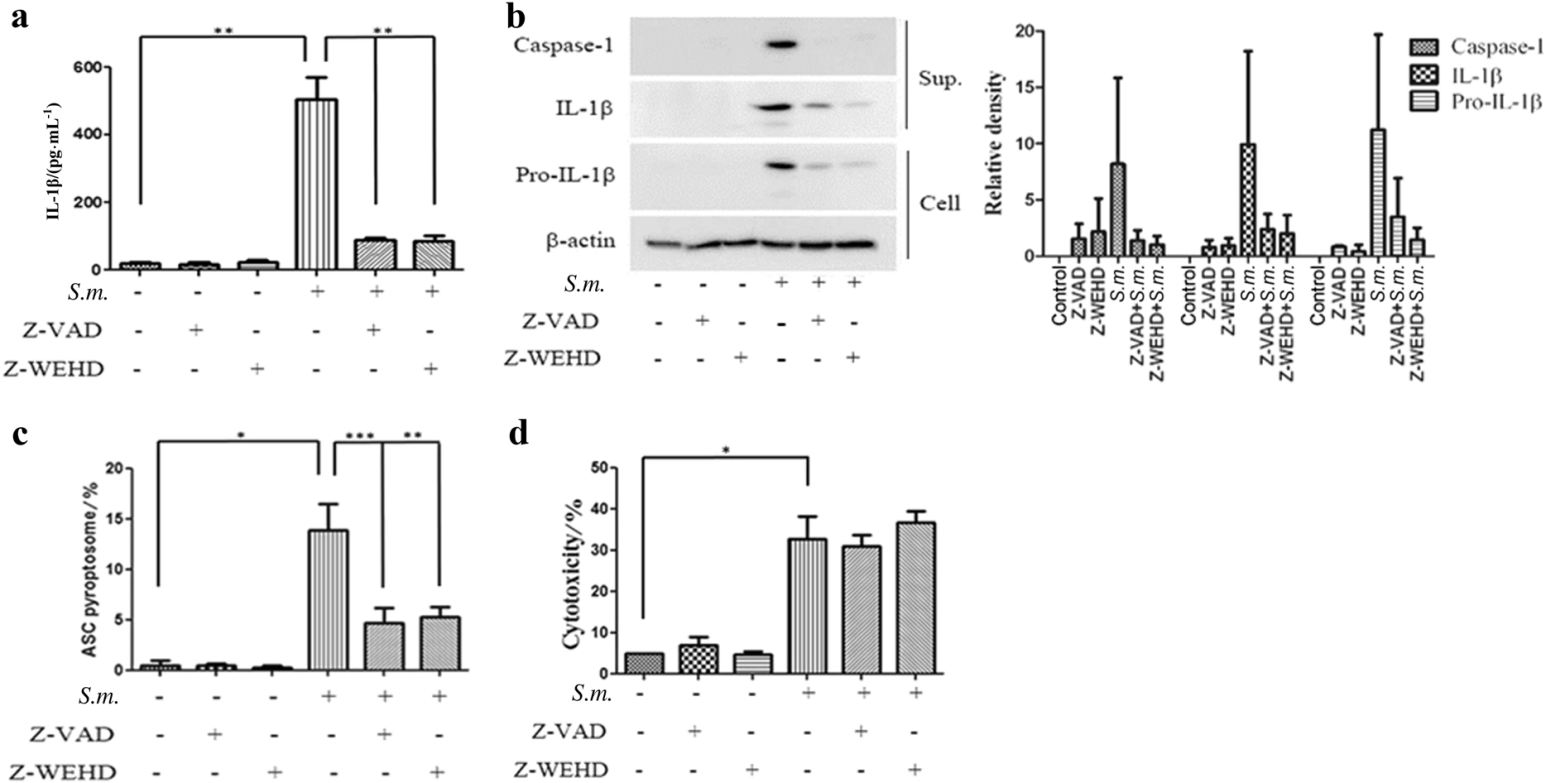
*S. mutans* induces caspase-1-dependent IL-1β secretion and caspase-independent cell death. **a**–**d** THP-1 cells were pretreated with Z-WEHD-FMK (10 μmol · L^−1^) or Z-VAD-FMK (10 μmol · L^−1^) for 30 min before *S. mutans* infection (MOI 50) for 24 h. **a** Cell culture supernatants were collected and assayed for IL-1β secretion by ELISA. **b** IL-1β and caspase-1 secreted into the culture supernatant (sup.) and pro-IL-1β and β-actin in the cell lysates (cell) were detected by immunoblotting. The relative western blot band densities were normalised to those of β-actin. **c** THP-1/ASC-GFP cells were pretreated with Z-WEHD-FMK or Z-VAD-FMK for 30 min before *S. mutans* infection for 24 h. The graph shows the percentage of cells containing ASC pyroptosomes. **d** Cell culture supernatants were collected, and the level of the cytoplasmic enzyme LDH was measured. The results represent those from one of three individual experiments. The data are reported as the means ± standard deviations (*n* = 3). *P < 0.05, **P < 0.01, ***P < 0.001. *S.m.* means *S. mutans*

To determine whether *S. mutans*-induced pyroptosome formation and cell death require caspase-1 activation, the cells were pretreated with the caspase inhibitors and infected with *S. mutans*. As a positive control, THP-1 cells were treated with *Escherichia coli(E.coli)* (Fig. S1). As shown in Fig. 2c, caspase inhibitors reduced ASC pyroptosome formation in response to *S. mutans* infection. On the other hand, LDH release was not blocked by the caspase inhibitors. These results suggest that *S. mutans*-induced ASC pyroptosome formation is dependent on caspase activation, but that cell death might not be mediated directly by caspase activation (Fig. 2d).

### *S. mutans* triggers IL-1β production via the NLRP3, AIM2 and NLRC4 inflammasomes

**Fig. 3:**
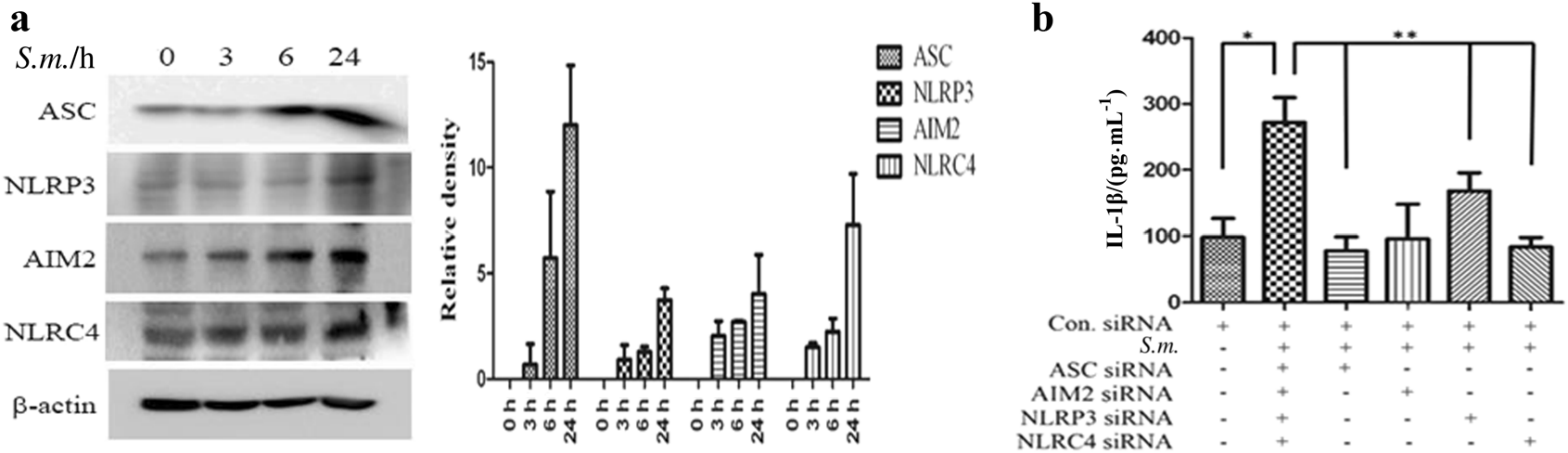
*S. mutans* infection induces NLRP3, AIM2, and NLRC4 inflammasome activation. **a** Immunoblot analysis of ASC, NLRP3, AIM2, NLRC4, and β-actin in *S. mutans*-infected cells at different time points. The relative western blot band densities were normalised to those of β-actin. **b** siRNA-transfected cells were infected with *S. mutans* (MOI 50) for 24 h. The cell culture supernatant was collected and assayed for IL-1β secretion by ELISA. The results represent those from one of three individual experiments. The data are reported as the means ± standard deviations (*n* = 3). *P < 0.05, **P < 0.01. *S.m.* means *S. mutans*

To examine the induction of inflammasome components by *S. mutans*, the protein expression of inflammasome components and IL-1β was determined by western blot analysis. *S. mutans* infection enhanced the expression of NLRP3, AIM2, and NLRC4 as well as the adaptor protein ASC. The level of each inflammasome component increased gradually over time (Fig. 3a).

To determine which inflammasome component is involved in *S. mutans*-induced IL-1β secretion, ASC, NLRP3, AIM2, and NLRC4 were knocked down individually by gene silencing in THP-1 cells. Treatment with each siRNA significantly blocked the expression of the respective protein (Fig. S2). When each inflammasome component was silenced, *S. mutans*-induced IL-1β secretion was significantly reduced, although silencing NLRP3 inhibited IL-1β secretion only slightly (Fig. 3b). Overall, these results suggest that the inflammasome components AIM2, NLRC4 and NLRP3 as well as ASC are involved in *S. mutans*-induced IL-1β secretion.

### Adenosine triphosphate (ATP) levels, potassium efflux and cathepsin B activity mediate *S. mutans*-induced IL-1β production

**Fig. 4:**
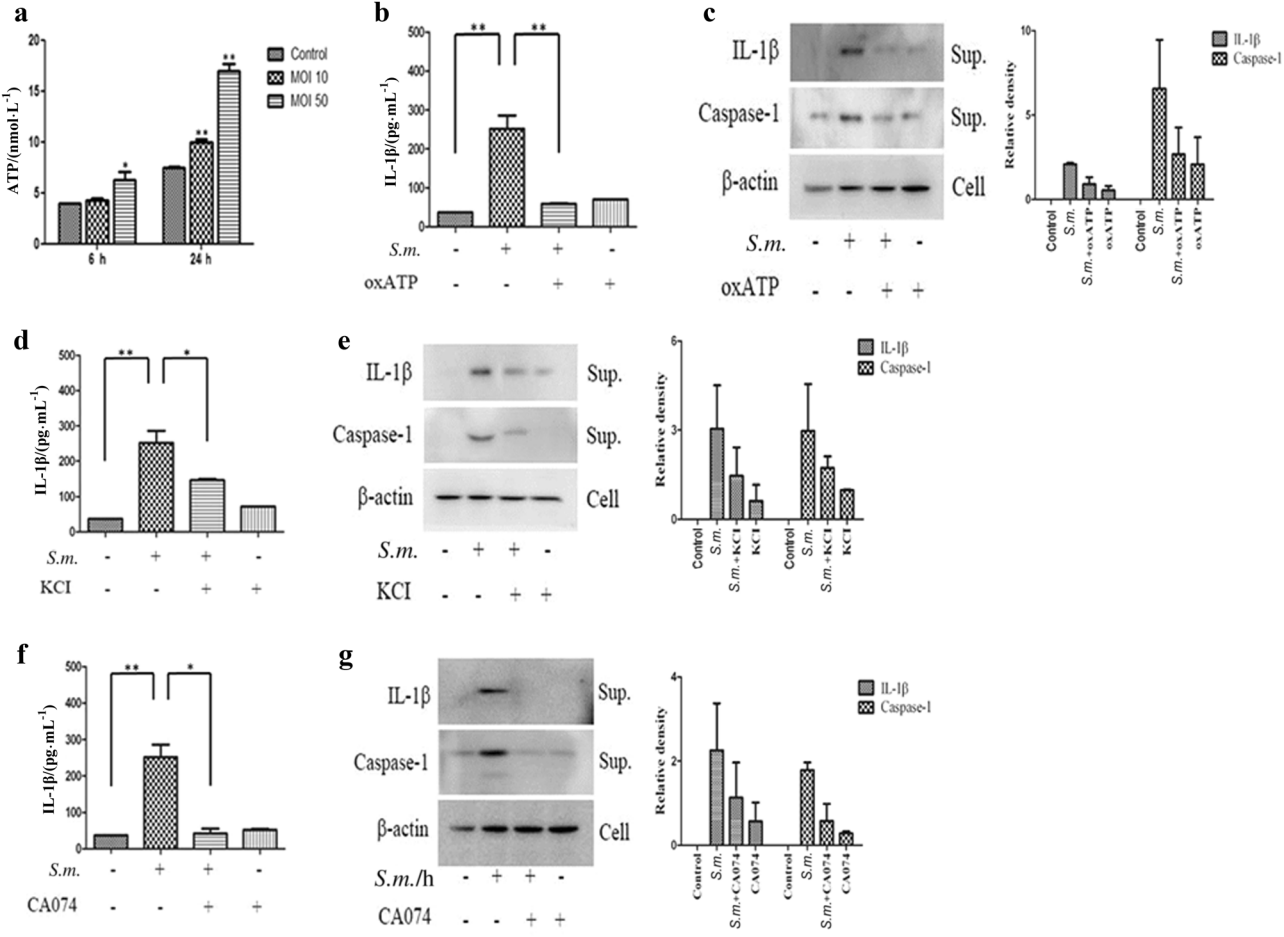
ATP release, potassium (K^+^) efflux, and cathepsin B activity are involved in *S. mutans*-induced NLRP3 activation. **a** THP-1 cells were infected with *S. mutans* (MOI 50) for 24 h. The extracellular ATP concentration was determined using an ATP determination kit. **b**–**g** THP-1 cells were pretreated with oxATP (100 μmol · L^−1^), KCl (500 μmol · L^−1^), or CA-074Me (5 μmol · L^−1^) for 30 min before *S. mutans* infection (MOI 50) for 24 h. Cell culture supernatants were collected and assayed for IL-1β secretion by ELISA (**b**, **d**, **f**). Secreted IL-1β and caspase-1 were detected in the culture supernatant (sup.) by immunoblotting (**c**, **e**, **g**). The results represent those from one of three individual experiments. The data are reported as the means ± standard deviations (*n* = 3). The relative western blot band densities were normalised to those of β-actin. *P < 0.05, **P < 0.01. *S.m.* means *S. mutans*

Although knockdown of NLRP3 had a smaller effect on the secretion of IL-1β than knockdown of the other inflammasome molecules, NLRP3 activation is important for bacterial infection-induced inflammation.^16^ Therefore, the NLRP3 inflammasome pathway was studied further. To confirm whether the NLRP3 inflammasome is critical for *S. mutans*-induced inflammation, the relationship between NLRP3 inflammasome mediators and *S. mutans*-induced IL-1β secretion was examined. First, the ATP concentration in the THP-1 cell culture supernatant after *S. mutans* infection was measured. *S. mutans* induced MOI- and time-dependent ATP release (Fig. 4a). Next, to determine whether the effects of *S. mutans* on caspase-1 activation and IL-1β secretion were mediated through ATP, the cells were pretreated with oxATP, a P2X7R antagonist that blocks the interaction of ATP with P2X7R. oxATP blocked the caspase-1 activation and IL-1β secretion induced by *S. mutans* (Fig. 4b, c). These results suggest that ATP mediates *S. mutans*-induced IL-1β secretion via NLRP3 inflammasome activation.

After stimulating the ATP-gated channel, the collapse of ionic gradients through K^+^ efflux occurs rapidly.^17,18^ Accordingly, this study examined whether K^+^ efflux also affects *S. mutans*-induced caspase-1 activation and IL-1β secretion. THP-1 cells were pretreated extracellularly with KCl for 30 min and then infected with *S. mutans*. As shown in Fig. 4d, e, *S. mutans*-induced caspase-1 activation and IL-1β secretion decreased significantly with increasing extracellular KCI concentrations. These results suggest that K^+^ efflux is needed for *S. mutans*-mediated NLRP3 inflammasome activation in THP-1 cells.

Various agents induce lysosomal damage and leakage of lysosomal proteases, specifically cathepsin B, into the cytosol, leading to NLRP3 inflammasome activation and IL-1β secretion.^19^ This study examined whether *S. mutans*-induced caspase-1 activation and IL-1β secretion are dependent on cathepsin B. THP-1 cells were pretreated with a cathepsin B inhibitor (CA-074Me) for 30 min and then infected with *S. mutans*. Inhibition of cathepsin B significantly blocked *S. mutans*-induced caspase-1 activation and IL-1β secretion (Fig. 4g). In addition, based on observation of the intracellular lysosomal distribution after *S. mutans* infection, *S. mutans* infection induced lysosomal activation near the bacteria, and inhibition of cathepsin B markedly reduced lysosomal activation (Supplementary Fig. S3). This evidence suggests that the effect of *S. mutans* on NLRP3 inflammasome activation is also mediated by lysosomal disruption.

Taken together, these results suggest that ATP levels, K^+^ efflux, and cathepsin B activity are critical factors in *S. mutans*-induced NLRP3 inflammasome activation and subsequent IL-1β secretion.

## Disscusion

The innate immune response plays a significant role in infections induced by pathogenic bacteria, including *S. mutans*. The initial interaction between bacteria and innate immune cells, such as macrophages and dendritic cells, is a critical event for the induction of the immune response to invading pathogens.^20^ This study examined the mechanism of IL-1β secretion in THP-1 cells infected with *S. mutans*. *S. mutans* induced caspase-1-dependent IL-1β secretion in THP-1 cells (Figs. 1 and 2). Caspase-1 is activated by a unique supramolecular complex called the pyroptosome, which is composed of oligomerized dimers of the adaptor protein ASC.^15^ The ASC complex, through assembly of a well-defined punctal structure in GFP-ASC-transduced macrophages, plays an important role in cellular homeostasis and caspase-1-dependent processing of cytokines.^21^ Several bacterial pathogens, such as *S. pneumoniae, B. abortus, S. typhimurium, P. gingivalis and A. actinomycetemcomitans*, induce the formation of ASC inflammasomes.^22^ This study showed that *S. mutans* infection induced ASC pyroptosome formation in THP-1 cells (Fig. 1c).

The expression of inflammasome sensor proteins, such as NLRP3, AIM2, and NLRC4, induced by *S. mutans* infection was examined. The AIM2 inflammasome is activated by detecting cytosolic DNA.^23^ *L. monocytogenes and F. tularensis* trigger AIM2 inflammasome activation.^24,25^ The NLRC4 inflammasome is activated mainly by microbial products such as bacterial flagellin and a conserved type three secretion system (TTSS).^12^ *Legionella pneumophila, Pseudomonas aeruginosa*, and *Shigella flexneri* stimulate NLRC4 inflammasome activation.^26^ The NLRP3 inflammasome is activated by diverse stimuli, including ATP, uric acid crystals, alum and pore-forming bacterial toxins.^27^ *Staphylococcus aureus, Listeria monocytogenes*, and *Neisseria gonorrhoeae* activate the NLRP3 inflammasome.^28^ In this study, the AIM2, NLRC4, and NLRP3 inflammasomes were activated in *S. mutans*-infected THP-1 cells. Similarly, a recent study reported that *Listeria monocytogenes* infection activates the NLRP3, NLRC4, and AIM2 inflammasomes.^8^ *L. monocytogenes* infection triggers multiple inflammasomes from NLRC4, which primes both AIM2 and NLRP3 inflammasomes, leading to caspase-1 activation. Regarding the NLRP3 inflammasome, this study examined whether ATP release, K^+^ depletion, and lysosomal disruption were involved in *S. mutans*-induced IL-1β secretion and caspase-1 activation. As expected, *S. mutans*-induced IL-1β secretion and caspase-1 activation were decreased by inhibition of *S. mutans*-induced ATP release, K^+^ depletion, and lysosomal disruption (Fig. 4). Overall, the *S. mutans*-induced inflammatory response was mediated by multiple inflammasome complexes, not a single type of inflammasome, and by ATP release, K^+^ depletion, and lysosomal disruption.

IL-1β synthesis, processing, and secretion are tightly controlled by a two-step mechanism.^29^ First, a transcriptional response results from nuclear factor-кB (NF-кB) activation, leading to the synthesis of pro-IL-1β.^30^ In parallel, a second signalling pathway activates caspase-1-dependent maturation and secretion of IL-1β via the inflammasome.^31^ *S. mutans*-induced IL-1β secretion was examined only by modulating the second signalling pathway.

Previous research has shown that *S. mutans*-induced IL-1β and TNF-α expression is mediated by the activation of NF-кB in RAW264.7 cells.^32^ NF-кB is a multiunit transcription factor that controls the induction of genes encoding proinflammatory cytokines.^33^ Many bacterial products and stimuli lead to NF-κB activation through innate Toll-like receptors (TLRs). TLRs sense invading pathogens outside the cell. TLR2 and TLR4, two members of the Toll-like receptor family, are expressed on the cell surface and act as signalling receptors to detect bacterial cell wall components. TLR2 recognizes a variety of bacterial components, such as lipoproteins and peptidoglycans.^34,35^ TLR4 functions as a signal transducer of lipopolysaccharide (LPS), a component of the outer membrane of Gram-negative bacteria.^34^ *S. mutans* increased TLR2 expression in THP-1 cells (data not shown). Upon activation, TLR signalling recruits a specific adaptor protein that harbours a Toll/interleukin-1 homology receptor (TIR) domain, such as myeloid differentiation primary response protein 88 (MyD88) or TIR-domain-containing adaptor-inducing interferon-β (TRIF), and initiates the downstream signalling transmission that leads to NF-кB activation, ultimately resulting in the upregulation of proinflammatory cytokines. Further study is needed to determine whether TLR is regulated by *S. mutans* infection and is involved in the transcriptional regulation of IL-1β via NF-кB activation. Such studies will lead to a better understanding of the molecular mechanisms of the *S. mutans*-induced inflammatory response.

Host cell death is an intrinsic immune defence mechanism in response to a microbial infection. On the other hand, bacterial pathogens use many strategies to manipulate the host cell death and survival pathways to enhance their replication and survival. These events are driven by the modulation of the mitochondrial pro-death (apoptosis), NF-κB–dependent pro-survival (autophagy), and inflammasome-dependent host cell death (pyroptosis) pathways during an infection. Understanding the bacterial pathogen-manipulated cell death pathways provides important insight into controlling infections.^36^ When THP-1 cells were stimulated with *S. mutans*, cell death was induced (Fig. 1d). In addition, no significant change in *S. mutans*-induced cell death was observed upon pretreatment with the caspase inhibitor Z-WHED-FMK or Z-VAD-FMK (Fig. 2d). This evidence indicates that *S. mutans*-induced cell death may not be associated with pyroptosis. Alternatively, *S. mutans* may induce different types of cell death, such as necrosis, apoptosis, and autophagy. Several researchers have reported that the families of the pathogen recognition receptor (PRR), such as TLRs and NLRs, appear to be related to host cell death. For example, induction of autophagy affects the TLR pathway.^35^ The involvement of TLRs or NLRs in the death of *S. mutans*-infected THP-1 cells should be examined further. Because we have mostly focused on elucidating the mechanism of inflammasome activation during *S. mutans* infection, factors or components that are involved in inducing inflammasome activation have not been studied. To confirm that bacterial immunomodulatory factors induce inflammation, we examined whether heat-inactivated *S. mutans* was capable of inducing inflammatory cytokines. Although heat-inactivated *S. mutans* did not show any cytotoxicity, heat-inactivated *S. mutans* induced IL-1β production in THP-1 cells similar to that induced by live *S. mutans* (Supplementary Fig. S4). Factors eliminated by heat inactivation did not have immunomodulatory ability. However, lipoteichoic acid (LTA) from *S. mutans* has been reported to induce TNF-α and NO in murine macrophages.^37^ Further studies are required to elucidate the bacterial component that induces inflammasome activation.

In summary, this study showed that infection with *S. mutans* triggered caspase-1-dependent IL-1β secretion via activation of the NLRP3, AIM2, and NLRC4 inflammasomes. In addition, *S. mutans*-induced NLRP3 inflammasome activation was mediated by ATP release, K^+^ depletion, and lysosomal disruption. This study offers novel insights into the innate immune response against *S. mutans* infection.

## Materials and methods

### Reagents

Phorbol 12-mystristate 13-acetate (PMA), oxidised ATP (oxATP), KCl, and trichloroacetate (TCA) were purchased from Sigma (St. Louis, MO, USA). CA-074 methyl ester (CA-074Me) was obtained from Calbiochem (San Diego, CA, USA). Z-VAD-FMK(pan-caspase inhibitor), Z-WEHD-FMK(caspase-1 inhibitor), anti-human IL-1β, and anti-human NLRP3 were supplied by R&D Systems (Minneapolis, MN). Anti-human ASC, anti-human AIM2, and anti-human caspase-1 antibodies were acquired from Cell Signaling Technology (Beverly, MA, USA). The anti-human NLRC4 and anti-β-actin antibodies were procured from Santa Cruz (Santa Cruz, CA, USA).

### Bacterial culture

*Streptococcus mutans* (strain Ingbritt, ATCC 25157) was cultured in brain-heart infusion (BHI) broth (BD, Franklin Lakes, USA) at 37 °C for 24 h. After incubation, viable *S. mutans* cells were counted according to an optical density at 650 nm (OD_650_) of 1.0 (equivalent to 2 × 10^8^ colony-forming units). *S. mutans* was harvested by centrifugation (Eppendorf, Hamburg, Germany) at 5000 rpm for 5 min, resuspended in RPMI-1640 medium (Gibco, Carlsbad, CA, USA), and used to infect the THP-1 cells at a MOI of 10 or 50.

### Cell treatment

Cells of the human acute monocytic leukaemia cell line THP-1 (ATCC TIB-202), were cultured in RPMI-1640 medium (Gibco, CA, USA) supplemented with 10% heat-inactivated foetal bovine serum (Gibco, CA, USA) at 37 °C in a 5% CO_2_ incubator. For the experiments, the cells were differentiated into macrophages using 50 ng·mL^−1^ PMA. The differentiated cells were infected with *S. mutans* for 45 min. The cells were then washed twice with PBS and incubated for 6 and 24 h in the presence of antibiotics (100 units per mL penicillin and 100 μg·mL^−1^ streptomycin) to kill the extracellular bacteria. The cells were pretreated with Z-WEHD-FMK, Z-VAD-FMK, oxATP, KCl, and CA-074Me at the indicated concentrations for 30 min before being infected with the bacteria.

### ASC pyroptosome quantitation in live cells

The THP-1/ASC-GFP cells were provided by Dr. SY Shin (University of Pohang, Republic of Korea). THP-1/ASC-GFP cells were seeded in 8-well chambers (Thermo, Rochester, NY, USA) and primed with PMA (50 ng · mL^−1^). The THP-1/ASC-GFP cells were challenged with *S. mutans*. ASC pyroptosome formation was observed using a confocal laser scanning microscope (LSM 700; Carl Zeiss, Jena, Germany). At least 400 cells were counted to determine the number of cells containing the ASC-GFP pyroptosome at each time point. The percentage of cells with an ASC pyroptosome was calculated by dividing the number of cells with an ASC pyroptosome by the total number of cells counted.

### Cytokine analysis

The amounts of IL-1β and TNF-α released into the culture media after *S. mutans* stimulation were analysed using an ELISA kit purchased from eBioscience (San Diego, CA, USA). The cytokine levels were measured according to the manufacturer’s instructions. The plates were read using an ELISA reader (Tecan, Männedorf, Switzerland) at 450 nm/570 nm.

### Immunoblot analysis

The whole-cell extracts were prepared by lysis of cells in RIPA buffer (10 mmol · L^−1^ Tris–HCl (pH 7.5), 150 mmol · L^−1^ NaCl, 1 mmol · L^−1^ ethylenediamine-N,N,N′,N′-tetraacetic acid, 1% NP-40, 0.1% sodium deoxycholate, and 0.1% sodium dodecyl sulfate) containing complete protease and phosphatase inhibitor cocktails (Roche, Penzberg, Germany). The protein in the culture supernatant was precipitated with 10% TCA (Sigma, St. Louis, MO, USA). The samples were subjected to sodium dodecyl sulfate-polyacrylamide gel electrophoresis and electrotransferred onto membranes (Millipore, Billerica, MA, USA). The membranes were incubated with the primary antibody, followed by incubation with a horseradish peroxidase-conjugated secondary antibody. The immunolabelled proteins were detected using an ECL kit (GE Healthcare, Piscataway, NJ, USA) and a LAS-4000 lumino-image analyser (Fuji Film, Tokyo, Japan). We used ImageJ software to quantify band densities relative to those of β-actin.

### Cell death assay

Cell death was measured with an LDH cytotoxicity assay kit (CytoTox96 non-radioactive cytotoxicity assay; Promega, Madison, WI, USA). Cytotoxicity was calculated using the following formula: percentage of cytotoxicity = 100 × (experimental LDH release – spontaneous LDH release) / (maximal LDH release – spontaneous LDH release). To determine the maximal LDH release, the cells were treated with 1% Triton X-100.

### ATP measurement

The extracellular ATP concentration was determined using an ATP quantification kit (Invitrogen, Carlsbad, CA, USA) according to the manufacturer’s instructions.

### RNA interference assay

Human small interfering RNAs (siRNAs) for ASC, NLRP3, AIM2, and NLRC4 and nontargeting control oligonucleotides were obtained from Qiagen (Valencia, CA, USA). The sequence IDs of each siRNA oligonucleotide used in this study are as follows: ASC siRNA, SI03086783; NLRP3 siRNA, SI03060323; NLRC4 siRNA, SI04374685; AIM2 siRNA, SI04261432; and nontargeting control siRNA, 1027281. For the siRNA experiments in THP-1 cells, the cells were seeded in 6-well plates at a density of 6x10^5^ cells per well in the presence of 50 nmol · L^−1^ PMA. The differentiated cells were then transfected with the siRNA oligonucleotides (1 200 ng) for 24 h using Attractene transfection reagent (Qiagen, Valencia, CA, USA) in 1 mL of RPMI-1640 medium.

### Statistics

The differences between samples were analysed using an unpaired, one-tailed Student’s *t* test. The data are reported as the means ± standard deviations, and *P* values of * < 0.05, ** <0.01 and *** < 0.001 were considered significant.

## Electronic supplementary material

Supplementary DataS1

Supplementary DataS4

Supplementary Data
