# Supplementary figures and images for: Streptococcus mutans activates the AIM2, NLRP3 and NLRC4 inflammasomes in human THP-1 macrophages
Source: Int J Oral Sci. 2018 Aug 6;10(3):23. doi: 10.1038/s41368-018-0024-z (PMC6080406; doi:10.1038/s41368-018-0024-z)

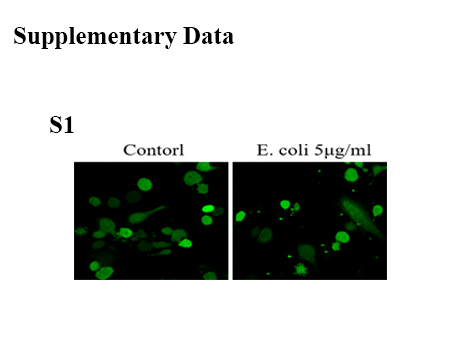

Supplement: Supplementary file 1 — Supplementary DataS1 [file 41368_2018_24_MOESM1_ESM.tif]

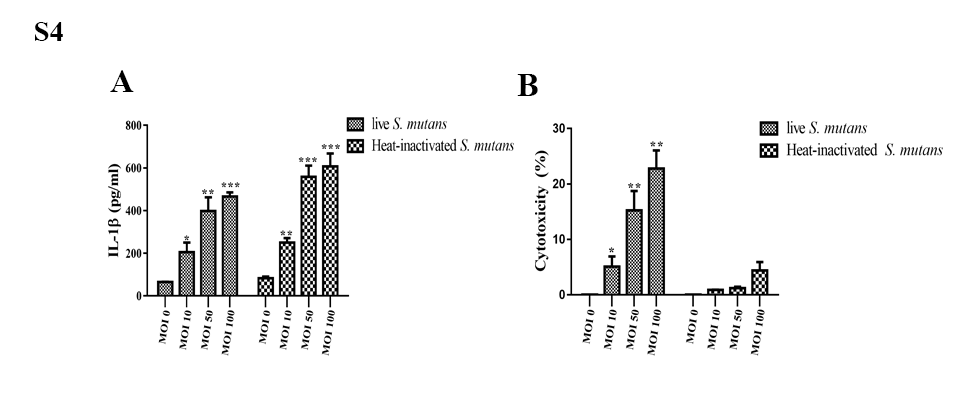

Supplement: Supplementary file 2 — Supplementary DataS4 [file 41368_2018_24_MOESM2_ESM.tif]

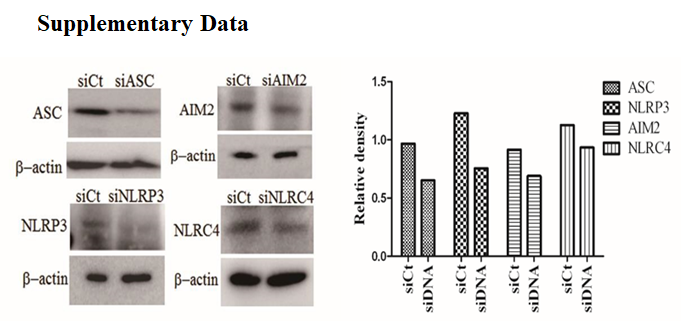

Supplement: Supplementary file 3 — Supplementary Data [file 41368_2018_24_MOESM3_ESM.tif]
